# DWPPI: A Deep Learning Approach for Predicting Protein–Protein Interactions in Plants Based on Multi-Source Information With a Large-Scale Biological Network

**DOI:** 10.3389/fbioe.2022.807522

**Published:** 2022-03-21

**Authors:** Jie Pan, Zhu-Hong You, Li-Ping Li, Wen-Zhun Huang, Jian-Xin Guo, Chang-Qing Yu, Li-Ping Wang, Zheng-Yang Zhao

**Affiliations:** ^1^ School of Information Engineering, Xijing University, Xi’an, China; ^2^ College of Grassland and Environment Science, Xinjiang Agricultural University, Urumqi, China

**Keywords:** plant, protein-protein interaction, network embedding, multi-source information, deep neural networks

## Abstract

The prediction of protein–protein interactions (PPIs) in plants is vital for probing the cell function. Although multiple high-throughput approaches in the biological domain have been developed to identify PPIs, with the increasing complexity of PPI network, these methods fall into laborious and time-consuming situations. Thus, it is essential to develop an effective and feasible computational method for the prediction of PPIs in plants. In this study, we present a network embedding-based method, called DWPPI, for predicting the interactions between different plant proteins based on multi-source information and combined with deep neural networks (DNN). The DWPPI model fuses the protein natural language sequence information (attribute information) and protein behavior information to represent plant proteins as feature vectors and finally sends these features to a deep learning–based classifier for prediction. To validate the prediction performance of DWPPI, we performed it on three model plant datasets: *Arabidopsis thaliana* (*A. thaliana*), mazie (*Zea mays*), and rice (*Oryza sativa*). The experimental results with the fivefold cross-validation technique demonstrated that DWPPI obtains great performance with the AUC (area under ROC curves) values of 0.9548, 0.9867, and 0.9213, respectively. To further verify the predictive capacity of DWPPI, we compared it with some different state-of-the-art machine learning classifiers. Moreover, case studies were performed with the AC149810.2_FGP003 protein. As a result, 14 of the top 20 PPI pairs identified by DWPPI with the highest scores were confirmed by the literature. These excellent results suggest that the DWPPI model can act as a promising tool for related plant molecular biology.

## Introduction

Prediction of protein–protein interactions (PPIs) in plants is of great biological importance (Fukao, 2012). Cells receive endogenous signals to regulate their gene expression under a special signaling pathway. In this process, proteins play an essential role in regulating and mediating the biological activities of plant cells (Lehti-Shiu and Shiu, 2012). In addition, the identification of PPIs not only helps understand how proteins perform their biological functions but also provides essential information for rational drug design. Traditional biological experimental methods, such as mass spectrometry (Woods et al., 2011), tandem affinity purification (Rohila et al., 2009), and yeast-two hybrid (Fang et al., 2002) were used. Nevertheless, these conventional approaches are costly, time-consuming, and prone to high false-positive rates. Thus, the development of novel computational models to identify potential PPIs would be of enormous value to plant genomics and genetics.

Recently, many bioinformatic methods have been proposed for identifying PPIs. These approaches can be roughly split into three categories: docking-based methods (Yan et al., 2017), structure-based methods (Hayashi et al., 2018), and sequence-based methods (Pan et al., 2021). Typically, the first two techniques perform better than the sequence-based methods. However, docking- and structure-based methods usually need the structural details of proteins. Problems arise when these prior data do not exist. Moreover, with the evolution of genome sequencing technology, a vast number of protein sequences have been discovered. Against this backdrop, the sequence-based approaches have attracted increasing attention. Most of the computational approaches adopt the machine learning algorithms such as support vector machine (Guo et al., 2008; Romero-Molina et al., 2019; Chakraborty et al., 2021), random forest (Li et al., 2012; Wang et al., 2019; Yang et al., 2020), and K-nearest neighbor (Ambert and Cohen, 2011; Ning et al., 2019). Some studies have also combined machine learning techniques with feature descriptors of protein sequences to predict PPIs, such as the Moran and Geary autocorrelation descriptor (Chen et al., 2020), conjoint Triad descriptor (Shen et al., 2007), and multi-scale local feature descriptors (You et al., 2015). These feature descriptors aim to summarize the information of 20 canonical amino acid sequences for PPI prediction.

Unlike the traditional machine learning approaches, deep learning-based approaches can not only extract high-dimensional features directly from the primary sequence (Ekbal et al., 2016; Zeng et al., 2020; Wang J. et al., 2021) but also capture their non-linear dependencies to increase prediction accuracy. Therefore, deep learning algorithms have been widely applied to predict associations between different biomolecules in recent years. For example, Czibula et al. (2021) introduced a method called AutoPPI to predict PPIs that used two autoencoders, which correspond to three kinds of neural network architectures. Qiang et al. (2020) presented an approach named CPPred-FL that used multiple feature descriptors to identify cell-penetrating peptides. CPPred-FL introduced a novel feature representation learning scheme to capture features from different perspectives. Huang et al. (2021) proposed a method, called MVMTMDA, for predicting microRNA–disease associations (MDAs). This model creates a multi-view representation of microRNAs that can predict MDAs via an end-to-end multitasking technique. Yuan et al. (2021) developed a deep graph-based framework named GraphPPIS for identifying PPIs. GraphPPIS transformed the prediction problem of PPI sites as a graph node classification task, which can be solved via deep learning techniques.

Recently, some studies have indicated that the information of network data is useful in prediction problems, including position, degree, and neighboring nodes in the graph. For example, Lim et al. (2019) presented a graph neural network, which used a distance-aware graph attention technique to predict drug-target interactions. Zhao et al. (2020) predict PPIs that combined the spatial relationship of protein sequence with the potential sequential feature of the ontological annotation semantics. Xu et al. (2020) developed a method called PPI-GE, which predicts PPIs by combining the contact graph energy and physicochemical graph energy. Xiao and Deng (2020) proposed a new node embedding approach to predict PPIs that captures the topological information from higher-order neighborhoods of PPI network nodes. Li et al. (2021) built a novel model called GAEMDA that used a graph neural network-based encoder to detect the miRNA-disease associations. Wang L. et al. (2021) presented a novel framework named NMFCDA to identify CircRNA-disease association by combining kernel similarity information, disease semantic information, and protein sequence information. Zheng et al. (2019) built a model called MLMDA to predict MDAs. This model combined miRNA functional similarity, Gaussian interaction profile kernel similarity information and disease semantic similarity with deep auto-encoder neural network and random forest classifier for the MDAs prediction. Guo et al. (2019) proposed a computational approach named LDASR to identify potential associations between lncRNAs and diseases. The method abstracted feature vectors for lncRNA and disease from multiple similarity matrices and the rotational forest algorithm is used for carrying the prediction.

Inspired by these graph embedding methods, we propose a novel efficient computational approach called DWPPI to predict potential protein–protein interactions in plants. This model employed two critical information: the original attribute information of the protein sequence, and the behavior information of the PPI graph network. To be specific, we first constructed a plants protein–protein bipartite graph to summarize the associations between these proteins, in which each plant protein is represented by a node, and each link represents their association. Then, we employed a graph embedding algorithm method, Deepwalk, to capture behavior information from the links, and used a word embedding algorithm, word2vec to encode the protein sequence for extracting attribute information. Thirdly, the behavior and attribute information were combined to form the fusion matrix, which is finally fed into a deep neural network (DNN) to predict potential plant-protein pairs. For evaluating the performance of the proposed method, we tested it on three model plant PPI datasets (including *Arabidopsis thaliana*, *Zea mays*, and *Oryza sativa*) based on fivefold cross validation (5-fold CV). As a result, DWPPI obtained 89.47, 95.00, and 85.63% prediction accuracy with the AUC of 0.9548, 0.9867, and 0.9213 on the three datasets, respectively. In comparison with different feature descriptors and machine learning-based classifiers, DWPPI also yields good predictive performance. Besides, we also tested a case study on the AC149810.2_FGP003 protein of the *Zea mays* dataset. Finally, 14 of the top 20 plant–protein interaction pairs with the highest prediction scores were confirmed in the published literature. These experimental results further demonstrated that our model brings new insights for discovering and exploring the intermolecular interactions.

## Results

### Evaluation Metrics

In this article, 5-fold CV was used to access the predictive performance of the DWPPI model. First, all the plant–protein pairs were randomly divided into five parts, which were disjoint and roughly equal. Second, four of the parts were used as the training set to train DWPPI, and the remaining one was adopted as the test set to yield the prediction results. Lastly, different sections were selected in turn as the training set, and step 2 was repeated until all sections were taken once and only once as the test set. The final experimental results were obtained by averaging the performance of five replicates. In this work, five parameters such as accuracy (Acc), Sensitivity (Sen), Specificity (Spec), Precision (PR.), and Matthew correlation coefficient (MCC) were performed to assess the predictive performance, which can be defined as:

$$ACC.=\frac{TN+TP}{TN+TP+FP+FN}$$ (1)

$$Sen.=\frac{TP}{FN+TP}\;$$ (2)

$$Spec.=\frac{TN}{FP+TN}$$ (3)

$$PR.=\frac{TP}{FP+TP}$$ (4)

$$MCC=\frac{TN\times TP-FN\times FP}{\sqrt{\mathrm{(ΤΝ+}FP\mathrm{)\times}\left(TP+FN\right)\mathrm{\times (}TN\times FN\mathrm{)\times (}TP+FP)}}$$ (5)

In the above formulas, TP,TN,FP , and FN represent the possible classification results including true positive, true negative, false positive, and false negative, respectively. The receiver operating characteristic (ROC) curves and precision-recall (PR) curves were adopted to evaluate the prediction ability of DWPPI. In addition, the area under ROC curves (AUC) was also computed to summarize the AUC value in a simpler way.

### Performance Evaluation Using Fivefold Cross Validation

**FIGURE 1:**
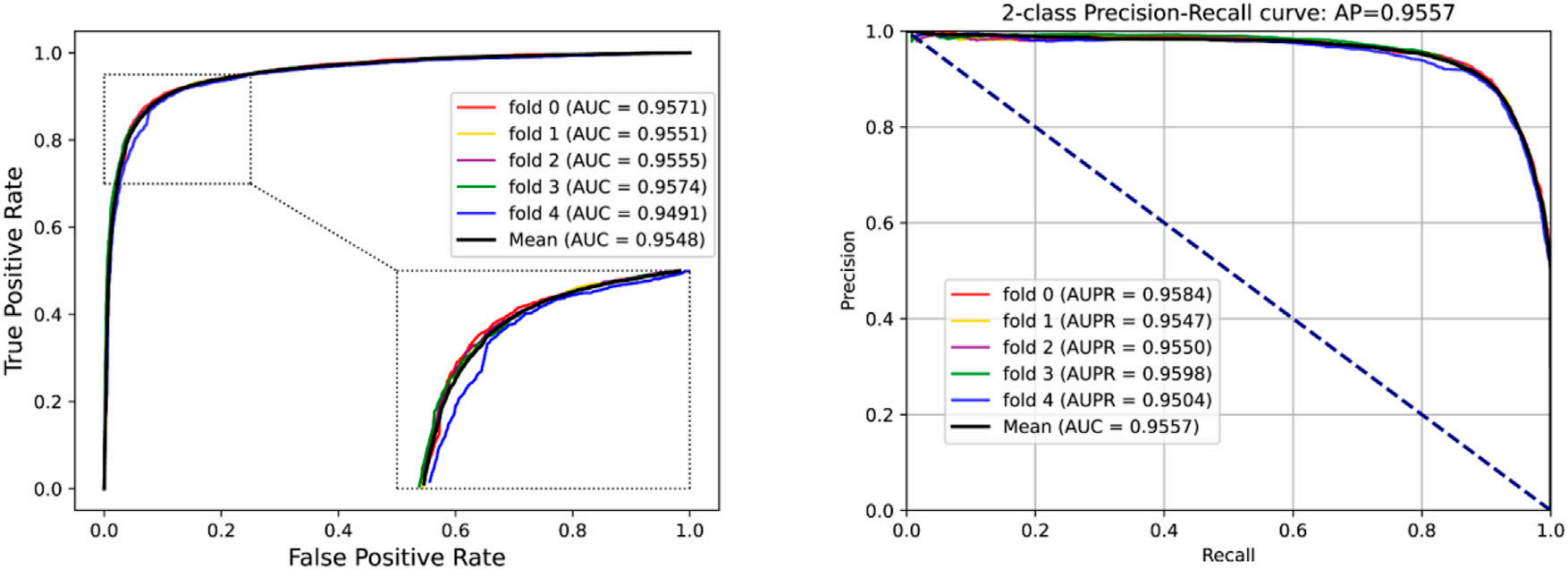
ROC and PR curves yielded by the DWPPI model on the *A. thaliana* dataset with the multiple feature.

**FIGURE 2:**
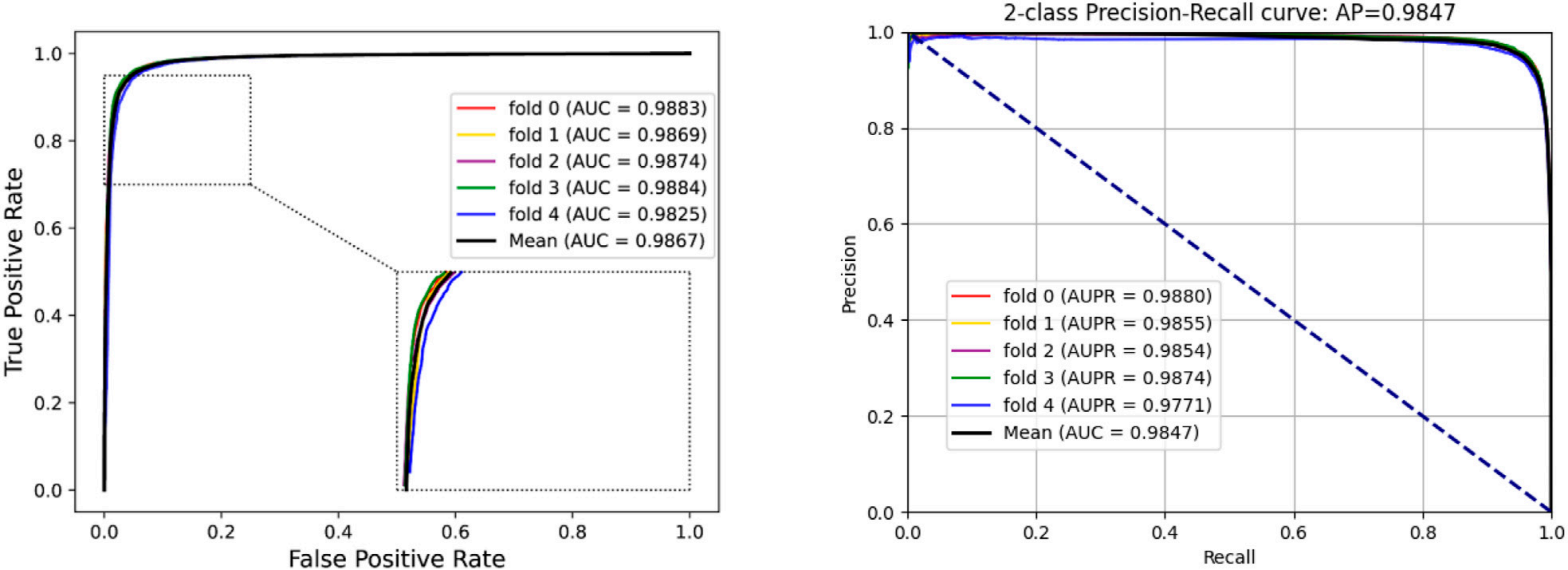
ROC and PR curves yielded by the DWPPI model on the *Zea mays* dataset with the multiple feature.

**FIGURE 3:**
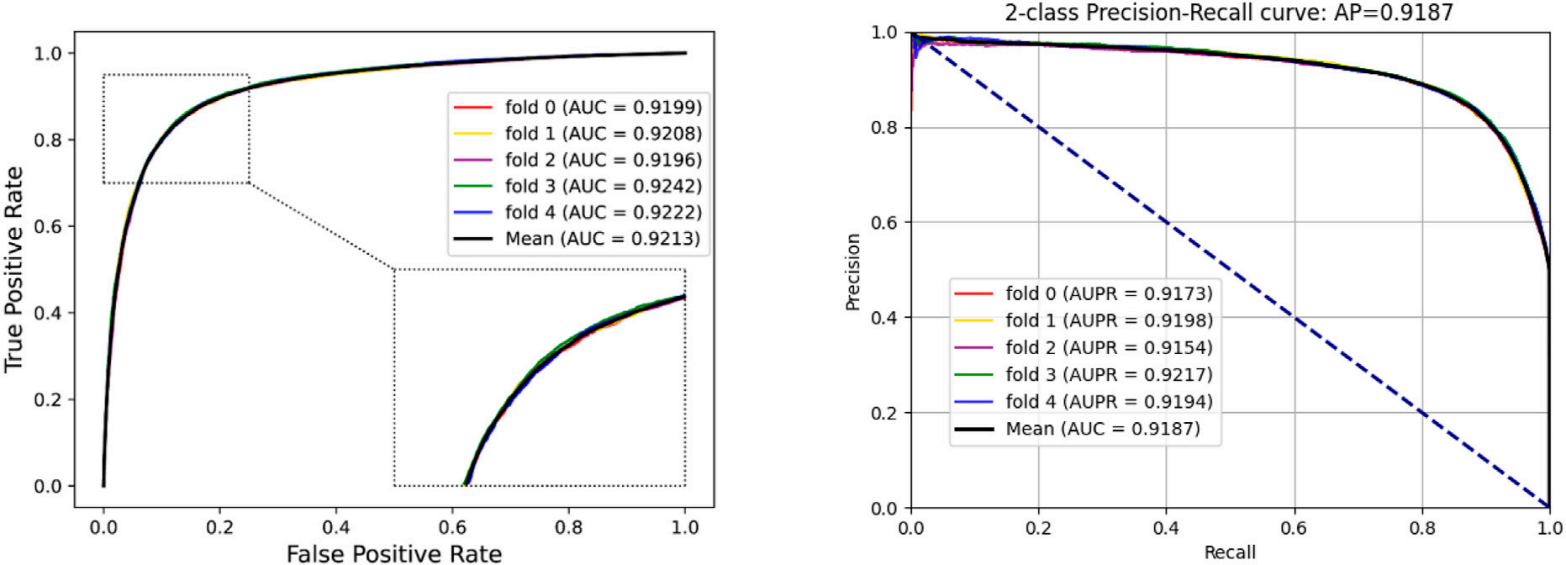
ROC and PR curves yielded by the DWPPI model on the *Oryza sativa* dataset with the multiple feature.

To access the capabilities of DWPPI, we performed it on the *A. thaliana*, *Zea mays*, and *Oryza sativa* datasets, respectively. To obtain better predictive stability and accuracy, we combined the behavior feature and attribute feature as the multiple feature to predict PPIs in plants. Table 1 summarizes the experimental results on the *A. thaliana* dataset, from which we can observe that the average ACC of fivefold CV method is 89.47%, the Sen is 91.47%, the Spec is 87.48%, the PR is 87.97%, the MCC is 79.02%, and AUC value is 0.9548, respectively. Their standard deviations are 0.32, 0.27, 0.88, 0.72, and 0.61% and 0.0034, respectively. Among the five sets of predictive performance, the lowest accuracy rate came to 88.97% and the best result rate of up to 89.85%. The experimental results of 5-fold CV on the *Zea mays* dataset are listed in Table 2. Here, it can be observed that the average ACC, Sen, Spec, PR, MCC, and AUC value obtained by DWPPI are 95.00, 96.30, 93.69, 93.85, 90.02% and 0.9867, respectively. The standard deviations are 0.38, 0.38, 0.70, 0.63, 0.75% and 0.0025, respectively. Table 3 lists the prediction results of the *Oryza sativa* dataset. The average ACC, Sen, Spec, PR, MCC and AUC value by 5-fold CV are 85.63, 86.38, 84.89, 85.11, 71.28%, and 0.9213, respectively. Their standard deviations are 0.17, 0.13, 0.23, 0.21, 0.35% and 0.0019, respectively. Figures 1–3 show the ROC and PR curves generated by the DWPPI model on the *A. thaliana*, *Zea mays*, and *Oryza sativa* PPI datasets, respectively.

**TABLE 1:** Prediction performance on the *A. thaliana* dataset with the multiple feature.

Testing set	ACC. (%)	Sen. (%)	Spec. (%)	PR. (%)	MCC. (%)	AUC
1	89.85	91.27	88.44	88.76	79.74	95.71
2	89.63	91.14	88.12	88.47	79.30	95.51
3	89.48	91.46	87.50	87.97	79.02	95.55
4	89.44	91.71	87.18	87.73	78.97	95.74
5	88.97	91.76	86.18	86.91	78.07	94.91
Average	89.47 ± 0.32	91.47 ± 0.27	87.48 ± 0.88	87.97 ± 0.72	79.02 ± 0.61	0.9548 ± 0.0034

**TABLE 2:** Prediction performance on the *Zea mays* dataset with the multiple feature.

Testing set	ACC. (%)	Sen. (%)	Spec. (%)	PR. (%)	MCC. (%)	AUC
1	95.38	96.49	94.28	94.4	90.79	98.83
2	94.94	95.98	93.9	94.02	89.90	98.69
3	94.94	95.87	94	94.11	89.89	98.74
4	95.30	96.79	93.8	93.98	90.63	98.84
5	94.42	96.35	92.48	92.76	88.90	98.25
Average	95.00 ± 0.38	96.30 ± 0.38	93.69 ± 0.70	93.85 ± 0.63	90.02 ± 0.75	0.9867 ± 0.0025

**TABLE 3:** Prediction performance on the *Oryza sativa* dataset with the multiple feature.

Testing set	ACC. (%)	Sen. (%)	Spec. (%)	PR. (%)	MCC. (%)	AUC
1	85.46	86.24	84.68	84.92	70.93	91.99
2	85.59	86.3	84.88	85.09	71.19	92.08
3	85.55	86.35	84.75	84.99	71.11	91.96
4	85.92	86.57	85.28	85.46	71.85	92.42
5	85.64	86.44	84.85	85.09	71.3	92.22
Average	85.63 ± 0.17	86.38 ± 0.13	84.89 ± 0.23	85.11 ± 0.21	71.28 ± 0.35	0.9213 ± 0.0019

### Performance Comparison of Different Classifiers on DWPPI

**FIGURE 4:**
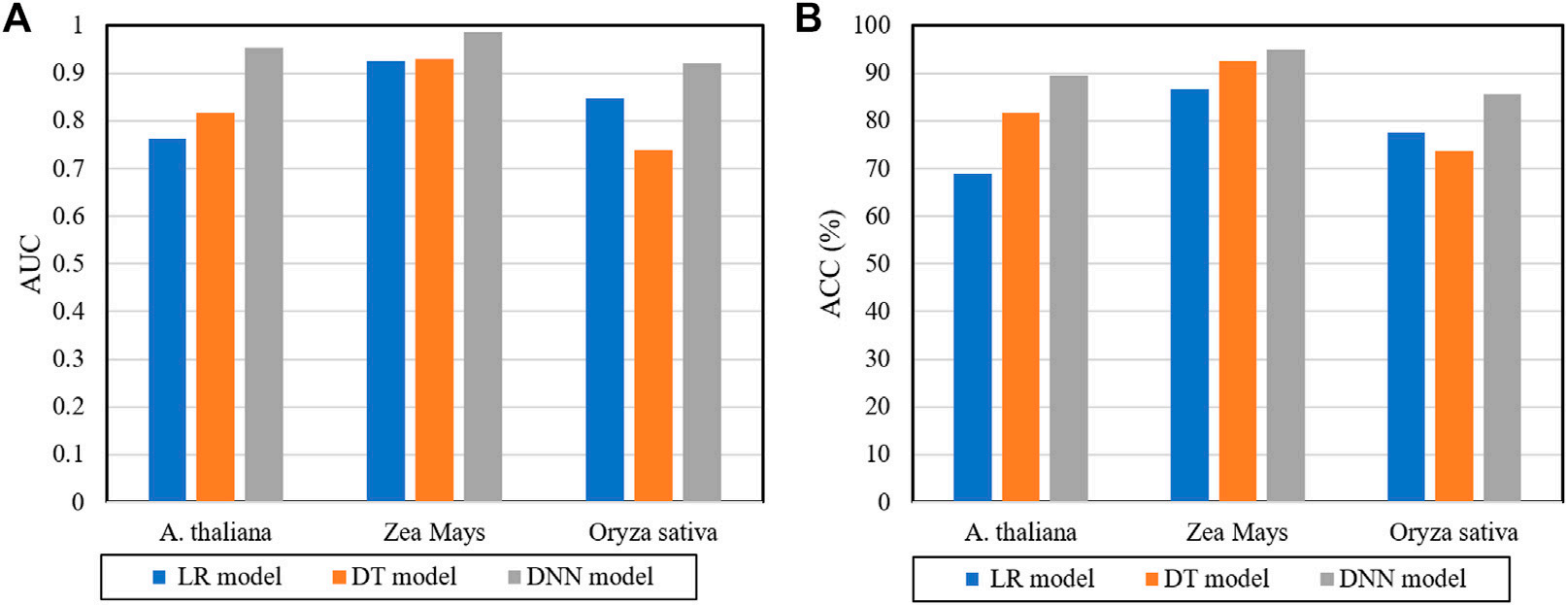
Comparison results of different classifiers with the DWPPI model **(A)** is the predicted AUC values of different classifiers on three plants PPIs datasets. **(B)** is the predicted ACC values of different classifiers on the three plants PPIs datasets.

In the prediction framework of the DWPPI model, we adopted the deep neural network (DNN) to classify the interaction between different plant proteins. In order to validate the effect of DNN on the DWPPI model, we made a comparison of the DNN model with some different classifier models. More concretely, we keep the multiple feature of the DWPPI model unchanged and experimented with some different classifiers instead of DNN, including logistic regression (LR) and decision trees (DT). The experimental results produced by these classifiers on the three plant PPI datasets are summarized in Table 4. It can be observed from Table 4 that the proposed model with the DNN as the classifier obtained significantly higher ACC and AUC values compared to other classifier models. For visual comparison, we present the ACC and AUC values as a histogram in Figure 4. These results indicated that DNN classifiers are applicable for the plant PPI prediction. The main reason for this performance is that the proposed deep learning framework can effectively mine the deep information embedded in the PPI network and significantly help increase the model performance.

**TABLE 4:** Comparison results of different classifiers in three model plant PPI datasets.

Plant	Classifier	ACC. (%)	Sen. (%)	PR. (%)	MCC. (%)	AUC
*A. thaliana*	LR	68.84	67.12	69.52	37.72	0.7639
	DT	81.81	81.92	81.74	63.62	0.8179
	Our method	89.47	91.47	87.97	79.02	0.9548
*Zea mays*	LR	86.70	85.63	87.49	73.41	0.9267
	Dt	92.60	92.78	92.44	85.20	0.9296
	Our method	95.00	96.30	93.85	90.02	0.9867
*Oryza sativa*	Loss	77.65	79.23	76.82	55.34	0.8476
	DT	73.78	70.07	74.62	47.58	0.7385
	Our method	85.63	86.38	85.11	71.28	0.9213

### Comparison of the Multiple Feature With the Attribute Feature and Behavior Feature

To further evaluate the efficiency of the proposed feature representation, we also performed experiments on the DNN model that only used the signal behavior or attribute feature via 5-fold CV. Table 5 provides the comparison results of the multiple feature with the feature that only used the behavior or attribute feature. In detail, the average predictive accuracy using the behavior information on *A. thaliana*, *Zea mays*, and *Oryza sativa* datasets was 82.33, 92.02, and 83.04%, and the yielded AUC values were 0.9078, 0.9627, and 0.9070, respectively. The average prediction results of using the attribute information on these datasets were 72.87, 90.41, and 80.41%; the obtained AUC values were 0.7632, 0.9476, and 0.8660, respectively. Taking the *A. thaliana* dataset as an example, the ACC gap between multiple and behavior features is 7.14%. Similarity, the ACC gap between multiple and attribute features is 16.6%. Compared with the results obtained by the multiple feature, we can conclude that employing the behavior or attribute feature alone cannot obtain better prediction results. All these experimental results demonstrated that the proposed multiple feature could help predict potential interaction between plant–protein pairs.

**TABLE 5:** Prediction performance on the three plant PPI dataset with different information.

Dataset	Feature	ACC. (%)	Sen. (%)	Spec. (%)	PR. (%)	MCC. (%)	AUC
*A. thaliana*	Behavior	82.33 ± 1.09	90.40 ± 1.38	74.16 ± 3.16	77.83 ± 1.99	65.48 ± 1.87	0.9078 ± 0.0088
	Attribute	72.87 ± 0.93	59.26 ± 4.16	86.48 ± 2.89	81.58 ± 2.28	47.63 ± 1.35	0.7632 ± 0.0048
	Multiple	89.47 ± 0.32	91.47 ± 0.27	87.48 ± 0.88	87.97 ± 0.72	79.02 ± 0.61	0.9548 ± 0.0034
*Zea mays*	Behavior	92.02 ± 0.43	93.61 ± 1.10	90.43 ± 0.91	90.73 ± 0.73	84.09 ± 0.86	0.9627 ± 0.0029
	Attribute	90.41 ± 0.77	91.07 ± 2.10	89.75 ± 0.74	89.89 ± 0.50	80.85 ± 1.59	0.9476 ± 0.0060
	Multiple	95.00 ± 0.38	96.30 ± 0.38	93.69 ± 0.70	93.85 ± 0.63	90.02 ± 0.75	0.9867 ± 0.0025
*Oryza sativa*	Behavior	83.04 ± 0.09	89.59 ± 1.00	76.49 ± 1.07	79.22 ± 0.57	66.67 ± 0.20	0.9070 ± 0.0035
	Attribute	80.41 ± 1.60	83.51 ± 2.98	77.32 ± 0.89	78.63 ± 0.92	60.97 ± 3.32	0.8660 ± 0.0209
	Multiple	85.63 ± 0.17	86.38 ± 0.13	84.89 ± 0.23	85.11 ± 0.21	71.28 ± 0.35	0.9213 ± 0.0019

### Case Study

To further evaluate the predictive ability of DWPPI, we performed a case study based on the *Zea mays* dataset. In the experiment, the AC149810.2_FGP003 protein was chosen to construct the case study, and all known protein–protein interactions provided by the *Zea mays* dataset were used to train DWPPI. The testing set was the PPI pairs consisting of the AC149810.2_FGP003 protein. After yielding the predicted results, we verified the top 20 PPI pairs with the highest predicted scores in the newly published literature. As shown in Table 6, 14 of the top 20 predicted proteins are verified in the experimental data provided by the PPIM dataset. The point to note is that the other six protein pairs of the unknown interaction are not proved by the literature, and there is no denying the possibility of interaction between them.

**TABLE 6:** Prediction of the top 14 predicted proteins based on known interactions on the *Zea mays* dataset.

Protein	Evidence	Protein	Evidence
GRMZM2G032222_P01	PPIM	AC193500.3_FGP003	PPIM
GRMZM2G068028_P01	PPIM	AC215639.3_FGP002	PPIM
AC209860.3_FGP004	unconfirmed	GRMZM2G143128_P01	unconfirmed
GRMZM2G069772_P01	PPIM	GRMZM2G147450_P01	unconfirmed
GRMZM2G072806_P01	PPIM	GRMZM2G013042_P01	PPIM
GRMZM2G125266_P01	unconfirmed	GRMZM2G013448_P04	PPIM
GRMZM2G096815_P01	PPIM	GRMZM2G172322_P01	unconfirmed
GRMZM2G141383_P01	unconfirmed	GRMZM2G020631_P01	PPIM
GRMZM2G000531_P03	PPIM	GRMZM2G026793_P01	PPIM
GRMZM2G004382_P01	PPIM	GRMZM2G020631_P01	PPIM

## Materials and Methods

### Data Collection

To evaluate the predictive performance of the DWPPI model, we applied it on three publicly available and widely used model plant datasets, *Arabidopsis thaliana* (*A. thaliana*), maize (*Zea mays*), and rice (*Oryza sativa*). Concretely, *A. thaliana* holds an esteemed position in the field of plant research and it makes a major contribution to the development of the plant protection, and increases the production of crops. The *A. thaliana* dataset was collected from public databases including IntAct (Kerrien et al., 2012), TAIR (Rhee et al., 2003), and BioGRID (Oughtred et al., 2019). After discarding the redundant PPIs, we yielded 28,110 PPI pairs from 7,437 different *A. thaliana* proteins. Although some negative sampling schemes had been developed previously, there is no single gold standard for constructing the non-interaction samples. The most widespread method is to select pairs randomly from non-interacted samples. The number of possible non-interaction pairs is 55,280,859 (7437 × 7437 − 28110), and we randomly selected 28,110 pairs as the negative samples for the *A. thaliana* dataset. Consequently, the whole *A. thaliana* dataset consisted of whole 56,220 protein pairs. We also tested DWPPI on the maize (*Zea mays*) and rice (*Oryza sativa*) datasets, which are two of world’s most economically important crops. For the *Zea mays* dataset, we collected 81,989 positive samples covering 7,199 different maize proteins from the PPIM database (Zhu et al., 2016). Similarly, we randomly selected 81,989 protein pairs from different subcellular localizations as the negative samples. Finally, *Oryza sativa* was constructed by 103,028 samples covering 3,760 types of rice proteins from the PRIN database (Gu et al., 2011). The number of proteins and interactions for these three model plant datasets are summarized in Table 7.

**TABLE 7:** Number of entries of the three different plant PPI datasets.

Plant	Protein number	Interaction number
*Arabidopsis thaliana* (*A. thaliana*)	7437	28110
*Zea mays* (Mazie)	7199	81989
*Oryza sativa* (Rice)	3760	51514

### Behavior Information

As a widely used graph-embedding approach, Deepwalk (Perozzi et al., 2014) was applied in the plant interaction network to represent the potential relationship of the vertices. In this work, let G represent the protein interaction network with group of vertices X and a set of edge Y , which is G=(X,Y) . Deepwalk consists of two main parts: 1) Random walk (RW), 2) the skip-gram algorithm (Mikolov et al., 2013b). The RW algorithm applies a random vertex $X_{j}$ to the graph G as the root of RW $W_{Xj}$ . In this part, we fixed the length t of the RW. Before reaching the maximum length t , the walk sequence will randomly choose the neighbors of the final passed node. For each sequence, the maximum co-occurrence probability of the vertices within T , and it can be defined as follows:

$$Pr\left(\left\{X_{j-w},\cdots ,v_{j+w}\right\}\backslash X_{j}|\phi \left(X_{j}\right)\right)=\Pi_{i=j-w,i\neq j}^{j+w}Pr\left(X_{i}|\phi \left(X_{j}\right)\right),$$ (6)

where j−w and j+w represent the left and right context of the word $X_{j}$ , T denotes the size of the window. Moreover, each vertex $X_{i}$ has been mapped to its current representation vector $\phi \left(X_{i}\right)\in R^{d}$ .

The skip-gram algorithm was used to iterate over all detected matches of the sequence in window T . For each j , $\phi \left(X_{j}\right)$ represents the vertex $X_{j}$ maps to the representation space; $\phi \in R^{\left|X\right|\times \sigma }$ is described as a matrix, the sample of all vertices is represented as |X| , and σ denotes the embedding size. After defining $X_{j}$ a representation in space, the probability of neighbors in the walk sequences should to be maximized. For convenience, we utilized the Hierarchical Softmax to factorize $Pr\left(X_{i}|\phi \left(X_{j}\right)\right)$ . The prediction tasks can be transformed as a hierarchy problem by assigning the vertices to the leaves of the binary tree. To accelerate the training time and maximize the specific path, the nodes of the Huffman tree can represent the vertices in the sequence. The $Pr\left(X_{i}|\phi \left(X_{j}\right)\right)$ can be expressed as follows:

$$pr\left(X_{i}|\phi \left(X_{j}\right)\right)=\overset{\left[\log\left|X\right|\right]}{\underset{k=1}{\Pi }}1/\left(1+e^{-\phi \left(X_{j}\right)•\varphi \left(b_{k}\right)}\right),$$ (7)

where $\varphi \left(b_{k}\right)\in R^{d}$ represents parent nodes of tree node $b_{k}$ . The sequence of tree nodes $\left(b_{0},b_{1},\ldots ,b_{\log\left|X\right|}\right)$ can be used to represent the path of $X_{i}$ , where $b_{\log\left|X\right|}=X_{i}$ , and $b_{0}=\text{root}$ . By allocating paths to frequent vertices in the RW, the Huffman tree that we adopted can accelerate the training process.

The embedding matrix ϕ could be yielded by applying the Deepwalk algorithm to the plant–protein interaction network. Each row of ϕ is a σ -dimensional vector, which will corresponded to a topological representation for each plant protein node. Thus, the cosine distance similarity between two vectors can be expressed as the similarity of two protein nodes $X_{i}$ and $X_{j}$ , which can be formulated as follows:

$$Sim\left(X_{i},X_{j}\right)=\frac{\sum_{k=1}^{\sigma }\phi \left(X_{i},k\right)\phi \left(X_{j},k\right)}{\sqrt{\sum_{k=1}^{\sigma }\phi {\left(X_{i},k\right)}^{2}}\sqrt{\sum_{k=1}^{\sigma }\phi {\left(X_{j},k\right)}^{2}}},$$ (8)

where $\phi \left(X_{i},k\right)$ and $\phi \left(X_{j},k\right)$ are the k-th components of the vector $\phi \left(X_{i}\right)$ and $\phi \left(X_{j}\right)$ . Based on Eq. 3, a topological similarity matrix $Sim_{P}$ can be built to represent the protein nodes in the PPI interaction network.

### Attribute Representation

In the DWPPI model, the word2vec algorithm (Mikolov et al., 2013a) was used to embed the protein sequence for capturing the attribute information of plant proteins. There are two main models in word2vec: 1) continuous bag-of-words model (CBOW) and 2) continuous skip-gram model (Skip-Gram). The difference between the CBOW and Skip-Gram model is that CBOW uses the context to predict the current words, while Skip-Gram applies the current word to predict the context. If the training data are not very big, the Skip-Gram method will be more efficient. In our experiment, considering the size of our plant PPI dataset, we selected the CBOW model of the word2vec algorithm to learn more frequent words and speed up the training time.

**FIGURE 5:**
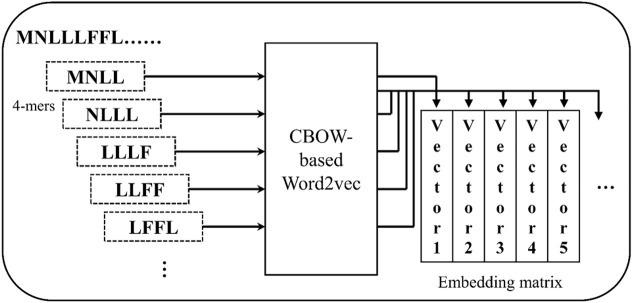
The framework of the word2vec model in the 4-mer case.

The amino acid sequences of these plant proteins were encoded as matrixes via the word2vec algorithm to extract the attribute information of plant nodes. The k-mers (k consecutive amino acids) method was used to regard the sequence as a word, and each protein sequence will be expressed as multiple k-mers. As shown in Figure 5, given a sequence MNLLLFFL, the unit of the 4-mers are MNLL, NLLL, LLLF, LLFF, and LFFL. To speed up the computation, the CBOW-based word2vec algorithm was selected to study the appearance pattern of the k-mers. Here, the protein sequences and k-mers correspond to the sentences and words in a natural language, respectively. In this work, the trained word2vec model will generate 64-dimensional embedding vectors in each k-mer to construct the embedding matrix of each protein. In the previous study, the 4-mer had been proved that it can achieve the optimal prediction accuracy via the 5-fold CV method.

### Deep Neural Network

Deep learning supports highly flexible architectures. In recent years, deep learning-based techniques have been widely used in the field of bioinformatics, such as recurrent neural network (RNN) (Kavuluru et al., 2017), deep belief network (DBN) (Wen et al., 2017), convolutional neural network (CNN) (Rifaioglu et al., 2020), and so on. Different deep learning architectures are appropriate for different problems. For example, RNN is suitable for exploring the sequential information, DBN is always used to account for high-dimensional correlations of biological data, and CNN is capable of extracting input complex features at different spatial scales (Lecun et al., 2015). Considering the interactions in plant proteins and the possible high dimension of behavior and attribute information, we used DNN as the architecture to predict potential PPIs in plants.

DNN is composed of an input layer, multiple hidden layers, and an output layer. Typically, the neural networks are fed data from the input layer, and then they will be transformed through the hidden layers in a non-linear way and the final result will be calculated to the output layer. The neurons in the hidden and output layers will be linked to all neurons in the previous layer. Each neuron computes a weighted sum of its inputs and utilizes a nonlinear activation function to derive its outputs P(x) (Angermueller et al., 2016). In this article, we selected the rectified linear unit (ReLU) (Xu et al., 2015) and sigmoid (Zhang and Woodland, 2016) as the activation function. In our experiments, we used the Deepwalk and word2vec algorithm to extract 64-dimensional behavior features and attribute features. Then, these two embedding matrices were fused together as the input data for the DNN. In order to accelerate the training process and avoid overfitting, the Adam algorithm (Kingma and Ba, 2014) and the dropout technique (Nair and Hinton, 2010) were also adopted in our model. We also employed cross-entropy as the loss function and ReLU as the activation function to get better results. Additionally, the batch normalization method was also used to speed up the training time and low sensitivity to initialization. The following equations can express the calculation of the loss:

$$T_{i1}^{m}=F_{1}\left(R_{i1}X_{i1}+b_{i1}\right)\left(i=1,\ldots ,n;m=1,2\right),$$ (9)

$$T_{ij}^{m}=F_{1}\left(R_{ij}T_{i\left(j-1\right)}+b_{ij}\right)\;\left(i=1,\cdots ,n;j=2,\cdots ,t_{1};m=1,2\right),$$ (10)

$$T_{ik}^{3}=F_{1}\left(R_{ik}\left(T_{it_{1}}^{1}⊕T_{it_{1}}^{2}\right)+b_{ik}\right)\;\left(i=1,\cdots ,n;k=t_{1}+1\right),$$ (11)

$$T_{ik}^{3}=F_{1}\left(R_{ik}T_{ik-1)}+b_{ik}\right)\;i=1,\cdots ,n;k=t_{1}+2,\cdots ,t_{2}),$$ (12)

$$L=-\frac{1}{n}\sum_{i=1}^{n}\left[\gamma_{i}\ln\left(F_{2}\left(R_{it2}T_{it2}+b_{it_{2}}\right)\right)+\left(1-\gamma_{i}\right)\ln\left(1-F_{2}\left(R_{it2}T_{it2}+b_{it2}\right)\right)\right],$$ (13)

where $h_{1}$ and $h_{2}$ represent the depth of individual and fused networks, n denotes the quantity of PPI pairs that need to be trained, and m indicates the individual network. Moreover, $F_{1}$ represents the ReLU function, $F_{2}$ denotes the sigmoid function, ⊕ is the concatenation operator, T is the output of hidden layer, and γ is the corresponding desired output. X is the batch training inputs, and R represents the weight matrix among the input and output layer, b is the bias.

## Conclusion

Predicting protein–protein interactions in plants help study the gene function of plans and also help understand essential roles thatthey play in a variety of biological processes. Systematically predicting potential plant–protein pairs will help increase crop yields. Compared to traditional wet experimental approaches, the dry experimental methods based on soft computing help analyze large-scale genetic data to detect new interactions between them. Thanks to the development of computing and storage capabilities of computers, the computational method helps quickly achieve scientific research results without the need for cell staining and pipettes. Moreover, the computational approaches effectively remove false positive signals, reduce unreliable results, and increase the chance of finding real but weak signals.

In this work, we used a natural language processing algorithm to describe the attribute information of protein nodes, and a graph embedding technique was used to represent the behavior information of protein links. Then, we combined the behavior and attribute information as the multiple feature to further improve the prediction power of the DWPPI model. The deep learning-based DNN classifier was adopted to train and predict these features. The presented DWPPI model integrates these algorithms organically and takes full advantage of their superiority, thus yielding excellent results in the experiment. In the 5-fold CV experiment, when performed on the model plant PPI datasets, *Arabidopsis thaliana*, *Zea mays*, and *Oryza sativa*, the proposed model obtains 89.47, 95.00, and 85.63% prediction accuracy with 0.9548, 0.9867, and 0.9213 AUC values, respectively. In further studies, we will investigate more natural language processing methods for problems of potential protein–protein interaction prediction in plants.

## Data Availability

Publicly available datasets were analyzed in this study. This data can be found here: Publicly available datasets were analyzed in this study. This data can be found here: http://arabidopsis.org/; http://www.ebi.ac.uk/intact; http://www.thebiogrid.org/; http://comp-sysbio.org/ppim; http://bis.zju.edu.cn/prin/.
